# Optical coherence tomography angiography and progressive retinal nerve fiber layer loss in mild to moderate open-angle glaucoma

**DOI:** 10.1371/journal.pone.0319903

**Published:** 2025-03-25

**Authors:** Zia Sultan Pradhan, Thanemozhi Srinivasan, Shruthi Sreenivasaiah, Dhanaraj AS Rao, Sujani Shroff, Sathi Devi, Carroll A B Webers, Harsha Laxmana Rao

**Affiliations:** 1 Department of glaucoma, Narayana Nethralaya Eye Hospital, Bangalore, Karnataka, India; 2 University Eye Clinic Maastricht, University Medical Center, Maastricht, the Netherlands; Seirei Hamamatsu General Hospital, JAPAN

## Abstract

**Purpose:**

To evaluate the association between optical coherence tomography (OCT) angiography measurements and progressive retinal nerve fiber layer (RNFL) loss in eyes with mild to moderate open-angle glaucoma.

**Methods:**

In this prospective study, 111 quadrants of 59 eyes with mild-moderate open-angle glaucoma were longitudinally evaluated for a minimum of 2 years with at least 3 OCT scans performed. OCT angiography was performed at baseline and vessel densities in the peripapillary and parafoveal regions were determined quadrant-wise. Effect of demographic (age, sex, presence of diabetes and hypertension), clinical (mean and fluctuation of intraocular pressure, presence of pseudoexfoliation, presence of disc hemorrhages, central corneal thickness, baseline mean deviation on visual fields) and OCT angiography parameters (peripapillary and parafoveal vessel densities) on rate of RNFL change was determined using linear mixed models.

**Results:**

Average baseline hemifield mean deviation and quadrant RNFL thickness was -3.5 ±  2.5dB and 97 ± 18µm respectively. The mean follow-up duration was 5.0 ±  1.8 years. The rate of change of quadrant RNFL thickness (RNFL slope) was -1.67 ±  0.2 µm/year. Multivariate mixed models showed that presence of pseudoexfoliation deposits (co-efficient -0.78 ±  0.34, p = 0.025) and optic disc hemorrhages (co-efficient -0.59 ±  0.28, p = 0.040) were associated with a faster rate of RNFL decline. None of the baseline OCT angiography parameters evaluated in this study showed any association with the RNFL slope.

**Conclusions:**

Presence of pseudoexfoliation and optic disc hemorrhages are significant risk factors for glaucoma progression in open-angle glaucoma. Baseline superficial vessel density, as measured on OCT angiography, was not associated with the rate of RNFL loss in mild-moderate open-angle glaucoma.

## Introduction

Glaucoma is a progressive, optic neuropathy and the identification of factors associated with disease progression is essential for its management. A meta-analysis of 50 population-based studies estimated that the worldwide prevalence of glaucoma in 2013 was 64.3 million which is expected to increase to 111.8 million by 2040 [1]. Since glaucoma is an irreversible, blinding disease, strategic designs for managing its screening and treatment are essential to reduce the economic burden of glaucoma. Patients with glaucoma who are at a greater risk of rapid progression need frequent follow-ups and more aggressive treatment [2]. Longitudinal population-based studies and prospective randomized clinical trials have identified some ocular risk factors for progression of open-angle glaucoma (OAG) such as higher intraocular pressure (IOP), pseudoexfoliation, presence of disc hemorrhages, and thinner central corneal thickness (CCT) [3,4]. Additionally, the Early Manifest Glaucoma Trial (EMGT) found certain systemic predictors of glaucoma progression, such as, lower systolic perfusion pressure and lower systolic blood pressure suggesting a vascular pathogenesis [4]. However, the association between local vascular factors within the eye and glaucoma progression is not clear.

In recent years, the development of optical coherence tomography angiography (OCTA) has enabled imaging of the vascular structures of the optic nerve head (ONH) and retina. Based on these images, several new parameters which are surrogate markers for the local, ocular perfusion, have been described and explored in glaucoma [5–7]. Some previous OCTA studies have found these vascular parameters to be predictive of glaucoma progression [6,8,9], while other studies did not find these parameters to have prognostic significance [10,11]. However, unlike the EMGT which included a range of OAG patients in its study cohort such as pseudoexfoliation glaucoma (PXG), normal-tension glaucoma (NTG) and primary open-angle glaucoma (POAG), most of these previous OCTA studies were exclusively on POAG. Hence, the purpose of the present study was to evaluate the association between OCTA measurements and progressive retinal nerve fiber layer (RNFL) loss in eyes with mild to moderate OAG including PXG, NTG and POAG.

## Materials and methods

This report is an analysis of participants recruited in a prospective, longitudinal study designed to evaluate glaucoma progression (Narayana Nethralaya Glaucoma Progression Study, NNGPS) conducted in Bengaluru, South India. These participants include glaucoma suspects and patients with different subtypes of glaucoma who are clinically evaluated every 6-12 months and undergo additional functional and imaging tests at these visits. The methodology adhered to the tenets of the Declaration of Helsinki for research involving human subjects. The Narayana Nethralaya Ethics Committee approved the study, and written informed consent was obtained from all participants.

For the purpose of this report, patients affected by glaucoma with open angles on gonioscopy (including POAG, NTG and PXG) with mild-moderate glaucoma at baseline, who visited the clinic between 17-09-2015 and 02-03-2023 were included. All participants underwent a comprehensive eye examination at baseline. This included a medical history, best-corrected visual acuity assessment, slit lamp biomicroscopy, IOP measurements on Goldmann applanation tonometry, gonioscopy, and dilated fundus examination. Exclusion criteria were age < 18 years, corrected distance visual acuity worse that 20/40, refractive error greater that ±  5 D sphere and ±  3 D cylinder, and any retinal or neurological disease that could confound evaluation. Additional investigations performed at this baseline visit included ultrasonic pachymetry (SP-3000, Tomey Corporation, Japan) for CCT measurements, visual field (VF) examination using Standard Automated Perimetry, Optical coherence tomography (OCT) imaging with Cirrus HD-OCT (Carl Zeiss Meditec Inc., Dublin, CA) and OCTA (Optovue Inc. Fremont, CA).

VF examination was performed using Humphrey Field Analyzer (Carl Zeiss Meditec Inc., Dublin, CA) with the Swedish interactive threshold algorithm (SITA) standard 24-2 program. VFs were considered reliable if false positive and false negative rates were < 15%. The VFs were considered glaucomatous if the glaucoma hemifield test was outside normal limits, pattern standard deviation was abnormal (p < 0.05) or ≥ 3 test points in a cluster were abnormal on the pattern deviation probability plot at p <  0.05 with at least 1 point abnormal at p <  0.01. Mean Deviation (MD) was used to quantify visual sensitivity loss and determine glaucoma severity. Since OCT is not ideal to detect progressive RNFL thinning in advanced glaucoma due to the measurement floor, only cases of mild-moderate glaucoma were included in the study. Since the glaucoma severity can be quite asymmetric between hemifields of the same eye and this is not evident from the overall MD, the MD of the superior and inferior hemifields were determined separately as provided on the Glaucoma Workplace 3.5 software (Carl Zeiss Meditec Inc. Dublin, CA). Only VF hemifields with mild-moderate glaucomatous damage (defined as hemifield MD better than -12dB) were included in the present analysis.

OCTA of the peripapillary and macular regions was performed at the baseline visit using Optovue SD-OCT (Angiovue, v2015.100.0.33). The procedure of OCTA imaging has been detailed previously [12–14]. In brief, the same location is repeatedly scanned and the variation in OCT signal caused by moving particles (such as red blood cells) is used to identify blood vessels. The split spectrum amplitude-decorrelation angiography (SSADA) algorithm is then used to delineate blood vessels. Vessel Density (VD) is defined as the area occupied by the large vessels and microvasculature in a particular region expressed as a percentage.

The optic disc OCTA scan is performed using volumetric scans covering an area of 4.5 ×  4.5 mm and the software automatically fits an ellipse to the optic disc margin. The peripapillary region is defined as a 0.75 mm-wide elliptical annulus extending from the optic disc boundary. This peripapillary region is divided into 8 sectors (temporal-upper TU, supero-temporal ST, supero-nasal SN, nasal-upper NU, nasal-lower NL, infero-nasal IN, infero-temporal IT, and temporal-lower TL). The average VD was calculated for the superior quadrant (ST and SN) and the inferior quadrant (IT and IN) and only those quadrants corresponding to the hemifields included were analyzed. The VD was calculated from the “Radial Peripapillary Capillary (RPC) segment” which extends from the internal limiting membrane (ILM) to the posterior boundary of the nerve fiber layer.

The macular OCTA scan is performed using volumetric scans covering a 3 x 3 mm area. The scan is divided by 2 circles (1 mm and 3 mm in diameter) centered on the fovea and the parafoveal region is defined as the annulus between these 2 circles. The parafoveal VD within the 90-degree quadrant (superior and/or inferior) corresponding to the hemifield included was analyzed. Parafoveal VD was analyzed from the superficial vascular plexus present between the ILM and the inner plexiform layer. Image quality was assessed for all OCTA scans and those with signal strength index < 35, motion artifacts or segmentation errors were excluded from the analysis.

OCT scanning was performed using Cirrus HD-OCT (Carl Zeiss Meditec Inc. Dublin, CA). The 200 x 200 optic disc cube scan protocol was performed. RNFL thickness was determined along a circle 3.46 mm in diameter positioned evenly around the center of the disc. Average RNFL thickness in the superior or inferior 90-degree quadrants corresponding to the hemifield included were analyzed. Image quality was assessed for all OCT scans and those with signal strength < 6, motion artifacts or segmentation errors were excluded from the analysis.

All participants were clinically evaluated every 6-12 months and OCT imaging was performed where possible. For the analysis of the current study, only those patients with mild-moderate glaucoma who were followed up for at least 2 years and had undergone a minimum of 3 OCT scans during their follow-up were included. Eyes that underwent any intraocular surgery (cataract or glaucoma surgery) during the study period were excluded from the analysis.

### Statistical analysis

Descriptive statistics included mean and standard deviation (SD) for continuous variables and percentages for categorical variables. The effect of demographic, clinical, VF, and OCTA parameters on rate of change of quadrant RNFL thickness (RNFL slope) was evaluated using linear mixed models with random intercepts and random slopes. In this model, the change in the outcome variable (quadrant RNFL thickness) was explored using a linear function of time, and random intercepts and random slopes introduced patient-, eye-, and quadrant-specific deviations from the average value. The model accounts for the fact that different quadrants and eyes can have different RNFL slopes over the follow-up period, while accommodating correlations between the 2 quadrants of an eye and/or both eyes of the same individual [15]. Because RNFL slope may depend on the disease severity, an unstructured covariance between random effects was assumed, allowing for correlation between intercepts and slopes [16]. Effects of predictor variables were assessed on the baseline RNFL thickness (baseline severity) and on the change in RNFL thickness over time by introducing interaction terms between time and predictor variables. The clinical parameters (predictors) investigated for their association with baseline RNFL thickness and rate of RNFL thickness change were age, sex, family history of glaucoma, presence of diabetes, hypertension, CCT, presence of a disc hemorrhage, presence of pseudoexfoliation deposits, follow-up duration, baseline IOP at time of recruitment for study, mean IOP and the IOP fluctuation (SD of IOP) during the follow-up period. The baseline VF, and OCTA predictors investigated were the hemifield MD, quadrant peripapillary VD, and quadrant parafoveal VD. Univariate models were constructed containing 1 predictor along with its interaction with time. Predictors associated with the rate of RNFL change at p <  0.10 in univariate analysis were introduced into multivariate analysis, along with other variables known to be risk factors for glaucoma progression. Parsimonious model building was performed to create a final model with the least number of independent variables. Collinearity between predictor variables was evaluated, and predictors that correlated with each other (correlation co- efficient of >  0.60) were evaluated in separate multivariate models. Rates of RNFL change were obtained from the linear mixed models using best linear unbiased prediction (BLUP) [17]. Statistical analyses were performed using Stata 14.2 software (StataCorp LLC). A p value of ≤ 0.05 was considered statistically significant for the final analysis.

## Results

One hundred and eleven hemifields of 59 eyes of 36 patients with OAG were included in the analysis. Table 1 provides the baseline demographic, clinical, VF, OCT and OCTA features of all included patients. The mean baseline age was 61.8 ±  8.0 years (range 41 to 79 years). Fourteen eyes (23.7%) had pseudoexfoliation deposits in the anterior segment. The mean hemifield MD at baseline was -3.5 ±  2.5dB. The mean RNFL thickness in the quadrant of interest was 97 ±  18µm at baseline and 89 ±  20µm at the final visit. The rate of change of quadrant RNFL thickness (RNFL slope) was -1.67 ±  0.2 µm/year.

**Table 1:** Characteristics of Patients with Open-angle glaucoma (111 hemifields from 59 eyes of 36 patients).

Variables	Data value
Age (years)	61.8 ± 8.0
Gender (male: female)	22: 14 patients
Positive family history of glaucoma (n, %)	3 patients (8.3%)
Diabetes mellitus (n, %)	15 patients (41.7%)
Hypertension (n, %)	22 patients (61.1%)
Spherical error (D)	0.4 ± 1.3
Presence of disc hemorrhage at baseline or during follow-up (n, %)	17 eyes (28.8%)
Presence of pseudoexfoliation deposits (n, %)	14 eyes (23.7%)
Baseline IOP at study recruitment (mmHg)	17.1 ± 5.0
Mean IOP during follow-up (mmHg)	15.2 ± 3.1
IOP fluctuation during follow-up (mmHg)	2.2 ± 0.9
Central Corneal Thickness (µm)	526 ± 34
Glaucoma medications at baseline visit	0.71 ± 0.9
Glaucoma medications at final visit	1.93 ± 0.9
Hemifields included (n = 111 hemifields)	
Superior	52
Inferior	59
Baseline Hemifield Mean Deviation (dB)	-3.5 ± 2.5
Baseline Quadrant RNFL Thickness (µm)	97 ± 18
Final Quadrant RNFL Thickness (µm)	89 ± 20
Baseline Quadrant Peripapillary vessel density (%)	57.7 ± 6.2
Baseline Quadrant Parafoveal vessel density (%)	47.6 ± 5.4
Follow-up duration (years)	5.0 ± 1.8
RNFL slope (µ/year)	-1.67 ± 0.22

IOP: intraocular pressure; dB: decibel; RNFL: retinal nerve fiber layer

All values represent Mean ±  Standard deviation for continuous variables and frequency (percentages) for categorical variables.

Table 2 summarizes the effect of each predictor on the baseline RNFL thickness and the RNFL slope in univariate analysis. A faster rate of RNFL loss (more negative slope) was associated with older age (coefficient =  -0.03, p = 0.04), presence of disc hemorrhage during follow-up (coefficient =  -0.50, p = 0.08), and presence of pseudoexfoliation deposits (coefficient -0.68, p = 0.05). The multivariate mixed-effect models are shown in Table 3. A model was built using these 3 parameters and other known risk factors for glaucoma progression such as mean IOP during follow-up and baseline MD. Then, parsimonious model building was performed in the multivariate analysis wherein the variables with the highest p values were eliminated in a step-wise manner to arrive at the final model. In this model, presence of pseudoexfoliation deposits (co-efficient =  -0.78, p = 0.025) and presence of disc hemorrhages (co-efficient = -0.59, p = 0.040) showed a significant association with the RNFL slope as highlighted in Table 3. On including average peripapillary VD into the multivariate model containing the above variables, no association was found between VD and RNFL slope (co-efficient =  0.05, p = 0.165). None of the baseline OCTA parameters evaluated in this study showed any association with the RNFL slope.

**Table 2:** Results of univariate analysis evaluating the effect of each predictive variable on the retinal nerve fiber layer (RNFL) in eyes with open-angle glaucoma.

Variable	Effect on Baseline RNFL thickness		Effect on RNFL slope	
	Co-efficient (Standard error)	P value	Co-efficient (Standard error)	P value^a^
Age	0.03 (0.3)	0.91	-0.03 (0.02)	**0.04**
Male gender	7.61 (5.1)	0.13	-0.31 (0.3)	0.26
Positive family history of glaucoma	-22.2 (8.2)	0.01	-0.08 (0.4)	0.83
Presence of diabetes	3.72 (5.1)	0.46	0.20 (0.27)	0.45
Presence of hypertension	-2.70 (5.2)	0.60	-0.05 (0.27)	0.84
Presence of disc hemorrhage	4.92 (4.3)	0.25	-0.50 (0.3)	**0.08**
Presence of pseudoexfoliation	2.71 (5.3)	0.61	-0.68 (0.35)	**0.05**
IOP at study recruitment	-0.90 (0.4)	0.05	0.006 (0.03)	0.82
Mean IOP during follow-up	-0.72 (0.8)	0.35	-0.01 (0.05)	0.79
IOP SD during follow-up	1.53 (2.5)	0.54	-0.16 (0.2)	0.40
Central Corneal Thickness	-0.03 (0.08)	0.69	0.003 (0.005)	0.54
Baseline hemifield Mean deviation	4.13 (0.5)	<0.001	-0.05 (0.06)	0.36
Baseline quadrant peripapillary VD	1.43 (0.2)	<0.001	0.01 (0.02)	0.52
Baseline quadrant parafoveal VD	0.25 (0.3)	0.50	0.01 (0.02)	0.54
Follow-up duration	-2.3 (1.2)	0.07	0.04 (0.1)	0.36

RNFL: retinal nerve fiber layer; IOP: intraocular pressure; SD: standard deviation; VD; vessel density.

^a^ P-value calculated using linear mixed models with random intercepts and random slopes.

**Table 3:** Results of multivariate mixed-effect models showing the factors associated with the slope of the retinal nerve fibre layer (RNFL) thickness in eyes with open-angle glaucoma. Values represent Co-efficient ±  Standard error with p value in parenthesis.

Variable	Model 1	Model 2	Model 3	Model 4
Age	-0.02 ± 0.02 (0.382)	-0.01 ± 0.02 (0.409)	–	–
Presence of disc hemorrhage	-0.58 ± 0.33 (0.076)	-0.59 ± 0.33 (0.072)	-0.70 ± 0.30 **(0.021)**	-0.59 ± 0.28 **(0.040)**
Presence of pseudoexfoliation	-0.71 ± 0.37 (0.054)	-0.71 ± 0.37 (0.055)	-0.81 ± 0.34 **(0.018)**	-0.78 ± 0.34 **(0.025)**
Mean intraocular pressure during follow-up	-0.05 ± 0.05 (0.323)	-0.05 ± 0.05 (0.287)	-0.05 ± 0.05 (0.274)	–
Baseline Mean Deviation	-0.04 ± 0.06 (0.448)	–	–	–

The patients were then divided into 2 cohorts, one containing PXG eyes only and another cohort including POAG and NTG eyes, and the analysis was repeated. As expected, the RNFL slope was greater in the PXG cohort (-2.2 ±  0.41 vs -1.54 ±  0.15 µm/year, p = 0.05). The univariate results were similar for both cohorts, that is, older age and the presence of disc hemorrhages were associated with faster RNFL progression in PXG and POAG. Baseline OCTA parameters did not have an association with RNFL slope in any of these glaucoma phenotypes.

Based on previous literature [9], to find a coefficient of 0.08 (standard error =  0.03) between baseline peripapillary vessel density and RNFL slope with an alpha error of 5%, the sample size of 111 hemifields had a power of 79.5%.

## Discussion

The current study was aimed at evaluating the association between baseline OCTA parameters and glaucoma progression in eyes with mild-to-moderate OAG and included patients with POAG, NTG and PXG. In contrast, previous studies have evaluated the role of OCTA in glaucoma progression only for POAG or NTG. We chose to explore these parameters in a mixed OAG cohort since these 3 conditions cannot always be differentiated in the clinic. The classification into NTG or POAG is dependent on the pre-treatment IOP (which is not always available) and other corneal parameters. Also, visualization of pseudoexfoliation deposits is not always possible in patients with PXG due to poorly dilating pupils or in eyes which have undergone cataract surgery and these patients may be misclassified as POAG. Hence, this study has explored the usefulness of baseline OCTA measurements as a risk factor for glaucoma progression in a real-world clinical setting.

In the present study, the baseline superficial vessel densities of the peripapillary and macular regions in OAG eyes of Asian-Indian ethnicity did not have any association with the progressive change in RNFL thickness over time. This finding is similar to a retrospective study of Korean OAG patients where the mean baseline circumpapillary capillary density was found to be similar in the patients that progressed (42.9 ±  5.7%) and those that did not progress (42.8 ±  6.5%, p = 0.99) [18]. Another study on Chinese participants with OAG or ocular hypertension also showed that the baseline superficial capillary density in the peripapillary and macular regions were not significantly associated with RNFL or VF progression [11]. In contrast, other studies on mild-moderate OAG have shown that lower baseline vessel density was associated with a significantly faster RNFL loss or VF progression [8,9,19]. However, none of these studies have included patients with PXG in their cohort. In the present study of patients with mild-moderate OAG which included POAG, NTG and PXG eyes, baseline OCTA parameters did not show any association with glaucoma progression.

In line with previous literature, the present study found that patients with PXG and eyes with disc hemorrhages had a higher risk of glaucoma progression [4,20]. Although these have been identified as risk factors in previous studies (prior to the development of OCTA technology), their persistent association with glaucoma progression after including newer OCTA variables such as vessel density into the analysis is an important finding. Also, since these risk factors are picked up on a simple, inexpensive slit-lamp examination, the present study re-iterates that a thorough clinical examination is of paramount importance in the follow-up of patients with glaucoma.

Increased IOP, which is an established risk factor for progression, was not found of be associated with RNFL slope in the present study [21]. One possible reason for this was that many patients included in this study were already started on treatment prior to being referred to our institute and hence baseline (pre-treatment) IOP was unavailable for several participants. Another possible reason for this could be the study design. In other studies, progression was defined based on an event-analysis and post-progression IOP values were not included in the analysis [21]. In contrast, in our study, progression was based on a trend-analysis of the RNFL slope and all IOP values during the study period were analyzed. Hence, in our study, neither the mean IOP nor the fluctuation in IOP during follow-up was found to be a risk factor for progression.

One of the strengths of the present study was the methodology used to study progressive RNFL decline. In the present study, the diagnostic criterion for mild-moderate glaucoma was based on the hemifield MD better than -12 dB and the criteria for progression was based on the RNFL slope in the superior or inferior quadrants on OCT. Considering global OCT measurements can mask localized RNFL change and several studies have shown that detecting glaucomatous progression on OCT with a region-of-interest approach (most typically seen in the superior or inferior quadrants) is better than global RNFL thickness measurements [22,23]. Therefore, the present study had the advantage of structural and vascular measurements being evaluated quadrant-wise.

The present study has some limitations. Each patient potentially contributed to 4 studied zones (2 quadrants each of 2 eyes) for glaucoma progression. Hence, some parameters were common to both eyes of a patient (demographic parameters like age, systemic illnesses) and some parameters were common to both quadrants of the same eye (presence of pseudoexfoliation, CCT, IOP). These issues were addressed by using appropriate statistical methods to analyze the data. Lastly, the time-interval between OCT scans to detect progression was not uniform and this could affect the RNFL slope estimation. This drawback was addressed by using BLUP instead of ordinary least square regression as it gives less weight to eyes with large variability and fewer measurements, hence, reducing the chance of bias.

## Conclusions

Baseline OCTA parameters, such as superficial vessel density, were not associated with the rate of RNFL loss in mild-moderate OAG. The presence of pseudoexfoliation and optic disc hemorrhages, which are clinical signs detected on a simple slit-lamp examination, were associated with faster glaucomatous progression. Hence, despite emerging technologies for monitoring glaucoma progression, a thorough clinical evaluation remains an integral part of the follow-up of patients with glaucoma.

## Supporting information

S1 Data Demographic, clinical, visual field and optical coherence tomography data of eyes with open-angle glaucoma .
(XLSX)
